# Risk factors for hepatic veno-occlusive disease caused by *Gynura segetum*: a retrospective study

**DOI:** 10.1186/s12876-018-0879-7

**Published:** 2018-10-26

**Authors:** Yan Wang, Dan Qiao, Ya Li, Feng Xu

**Affiliations:** 1 Department of Gastroenterology, The First Affiliated Hospital of Zhengzhou University, 1 Jianshe Donglu, Zhengzhou, 450052 Henan China; 2 Department of Zhengzhou Center for Disease Control and Prevention, Zhengzhou, China

**Keywords:** Hepatic veno-occlusive disease, *Gynura segetum*, Anticoagulant, Transjugular intrahepatic portosystemic shunt, Prognostic factor

## Abstract

**Background:**

Hepatic veno-occlusive disease (HVOD) caused by *Gynura segetum* has been increasingly reported in China in recent years. The aim of this retrospective study was to identify independent prognostic markers for survival in patients with *Gynura segetum-*induced HVOD and to evaluate the effect of anticoagulants and transjugular intrahepatic portosystemic shunt (TIPS) on survival rate.

**Methods:**

Clinical data including symptoms, signs, imaging characteristics, laboratory test results, results of liver tissue biopsies, type of treatment during follow-up and clinical outcomes were collected. Univariate, multivariate and time-dependent Cox regression analyses were performed.

**Results:**

Survival rates were 91% (95% confidence interval [CI], 82–95%), 64% (95% CI, 53–69%) and 57% (95% CI, 51–65%) at 1, 3 and 60 months, respectively. Total bilirubin, albumin and hepatic encephalopathy were independent prognostic markers of survival. Anticoagulants were administered to 76% of the patients. Among 75 patients treated with anticoagulants, 49 patients (65.3%) were cured, whereas 26 patients (34.7%) died; the cure rate in anticoagulant-treated patients was higher than that of those not treated with anticoagulants (χ^2^ = 9.129, *P* = 0.004). Cure rate of the anticoagulation + TIPS treatment group was 64.3%, which was also higher than that of the non-anticoagulation group; however, this was not significantly different (χ^2^ = 3.938, *P* = 0.096).

**Conclusions:**

The presence of hepatic encephalopathy, serum bilirubin and albumin levels were major prognostic factors for *Gynura segetum*-induced HVOD. Anticoagulation therapy significantly increased the cure rate; however, TIPS treatment did not have a beneficial effect on the cure rate.

## Background

Hepatic veno-occlusive disease (HVOD), also termed hepatic sinusoidal obstruction syndrome, is a rare clinical syndrome caused by several factors including high-dose chemotherapy before haematopoietic stem cell transplantation (HSCT) [1, 2], high-dose chemotherapy and ingestion of herbal compounds containing pyrrolizidine alkaloids (PAs) [3]. In China, HVOD has rarely been reported in patients who received HSCT despite the large number of HSCT cases. HVOD caused by *Gynura segetum* (i.e. Tusanqi)-containing PAs, a Chinese medicinal herb used for self-medication as well as pain relief, hypertension and dissipation of blood stasis [4], has been increasingly reported in China in recent years [5–7].

HVOD is defined as intrahepatic post-sinusoidal portal hypertension caused by stenosis or occlusion of veins, including the central veins of hepatic lobules and the sublobular veins [8]. HVOD is associated with significant mortality due to the severity of the disease and the absence of uniformly effective therapies. Until recently, treatment approaches have largely involved supportive and symptomatic care, such as restriction of water and sodium intake, diuretics, paracentesis and albumin infusion. Other treatment options include anticoagulant therapy, transjugular intrahepatic portosystemic shunt (TIPS) [9] and liver transplantation [10].

HSCT-related HVOD differs from that associated with *Gynura segetum* in many aspects, such as aetiology, ethnicity, underlying diseases, clinical findings and treatment. Not much is known about factors that might be relevant in predicting survival in patients with *Gynura segetum*-related HVOD. Most studies are case reports or include limited numbers of patients because of the rarity of this clinical presentation. The aim of this retrospective study was to identify independent prognostic markers for survival in patients with *Gynura segetum*-induced HVOD and to evaluate the effect of anticoagulants and TIPS on survival.

## Methods

### Patients and database

Between July 2012 and July 2017, 132 patients admitted to the First Affiliated Hospital of Zhengzhou University with a clinical diagnosis of HVOD and stated to have ingested *Gynura segetum* were included in this retrospective study. Previously described diagnostic criteria for Tusanqi-induced HVOD was used [11–13]. Patients with the following conditions were excluded from the study: chronic liver disease due to other causes, hepatocellular carcinoma, Budd–Chiari syndrome, congestive heart disease and incomplete data.

This study was approved by the Ethics Committee of the First Affiliated Hospital of Zhengzhou University in Zhengzhou, China. Written informed consent to participate was obtained from all patients. In all cases, the first available data were used as baseline data. Clinical data including symptoms, signs, imaging characteristics, laboratory test results, results of liver tissue biopsies, types of treatment during follow-up and clinical outcomes were collected. All patients were followed up from the date of diagnosis until death, lost to follow-up or study closure on 31 July 2017.

### Treatment

Patients who received treatments other than anticoagulants, such as diuretics, paracentesis, albumin and/or liver unction protection, were categorised in the non-anticoagulation group. After exclusion of contraindications, anticoagulation therapy was administered as follows: oral warfarin at 1.5 mg daily, with dose adjustment to maintain an international normalised ratio between 2 and 3, combined with low-molecular-weight heparin at 4000 IU by subcutaneous injection every 12 h. In patients whose symptoms improved, anticoagulation therapy was maintained until complete remission. In patients whose symptoms did not improve or worsened after 2 weeks of anticoagulation therapy, TIPS was performed following assessment of the patient.

### Statistical analysis

All statistical analyses were performed using SPSS statistical software version 17.0 (SPSS, Chicago, Illinois, USA). All continuous variables were expressed as medians (25th–75th percentiles) and compared with Student’s *t* test or non-parametric test according to the distribution characteristics. Categorical variables were expressed as numbers with percentages and compared by the χ^2^ test or Fisher’s exact test. Survival rates were calculated using the Kaplan–Meier method. Univariate survival analysis to assess the effect of patient characteristics was based on comparison of survival curves by the log-rank test. Statistically significant variables were introduced into a multivariate Cox’s proportional hazards analysis. A *P* value less than 0.05 was considered to indicate a significant difference.

## Results

### Patient characteristics

**Fig. 1:**
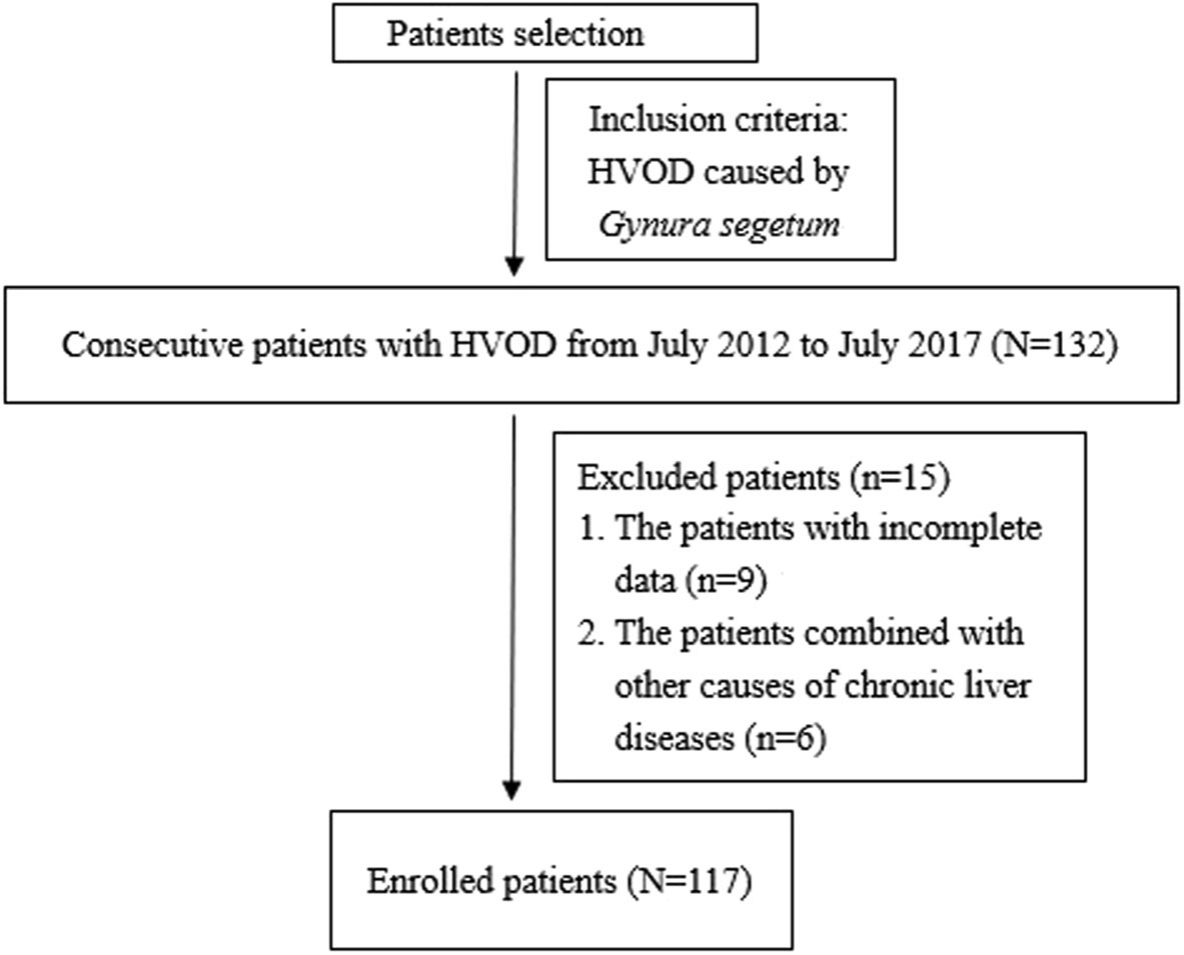
Flowchart of patient selection, inclusion, and exclusion

After the exclusion of 15 patients who did not fulfil the inclusion criteria, a total of 117 patients who were eligible were included in the analysis (Fig. 1). Patient characteristics at the time of diagnosis are presented in Table 1. Median age was 63 years (range, 18–84 years), and 67% of the patients were male. The most common clinical presentation was ascites (99.1%), followed by abdominal distention (98.3%), hepatomegaly (70%), poor appetite (41%), lower limb oedema (39.3%), splenomegaly (34.2%) and malaise (33.3%).

**Table 1:** Characteristics of 117 patients with hepatic veno-occlusive disease at the time of diagnosis

Characteristic	Obtained data
Age (years)	63.0 (52.5–69.0)
Male/female (%)	67/33
Clinical manifestations, n (%)	
Ascites	116 (99.1)
Abdominal distention	115 (98.3)
Poor appetite	48 (41.0)
Nausea, vomiting	19 (16.2)
Malaise	39 (33.3)
Lower limb oedema	46 (39.3)
Hepatomegaly	82 (70.0)
Splenomegaly	40 (34.2)
Right upper quadrant pain	23 (19.7)
Weight gain	21 (18.0)
Encephalopathy	19 (16.2)
Variceal bleeding	3 (2.6)
Laboratory tests	
WBC (10^9^/L)	6.1 (5.0–8.7)
RBC (10^12^/L)	4.4 (3.9–4.9)
PLT (10^9^/L)	113 (78–153)
ALT (U/L)	49.0 (25.0–152.5)
AST (U/L)	75.0 (39.0–158.5)
GGT (U/L)	100.7 (61.8–164.8)
ALP (U/L)	122.0 (86.8–191.3)
TB (μmol/L)	33.3 (19.7–47.0)
DB (μmol/L)	21.5 (12.7–30.5)
ALB (g/L)	30.6 (27.7–33.6)
PT (s)	14.8 (12.7–17.0)
BUN (mmol/L)	5.5 (3.8–7.9)
CR (μmol/L)	74.0 (60.0–90.0)
CA-125 (U/ml)	259.9 (182.1–458.4)
CA199	13.04 (7.9–34.2)
Treatment, n (%)	
Non-anticoagulation	26 (22.2)
Anticoagulation	74 (63.2)
Anticoagulation + TIPS	17 (14.5)

*WBC* white blood cell count, *RBC* red blood cell count, *PLT* platelet count, *AST* aspartate aminotransferase, *ALT* alanine aminotransferase, *GGT* λ-glutamyl transferase, *ALP* alkaline phosphatase, *TB* total bilirubin, *DB* direct bilirubin, *TP* total protein, *ALB* albumin, *BUN* blood urea nitrogen, *CR* creatinine, *PT* prothrombin time, *TIPS* transjugular intrahepatic portosystemic shunt

### Survival

**Fig. 2:**
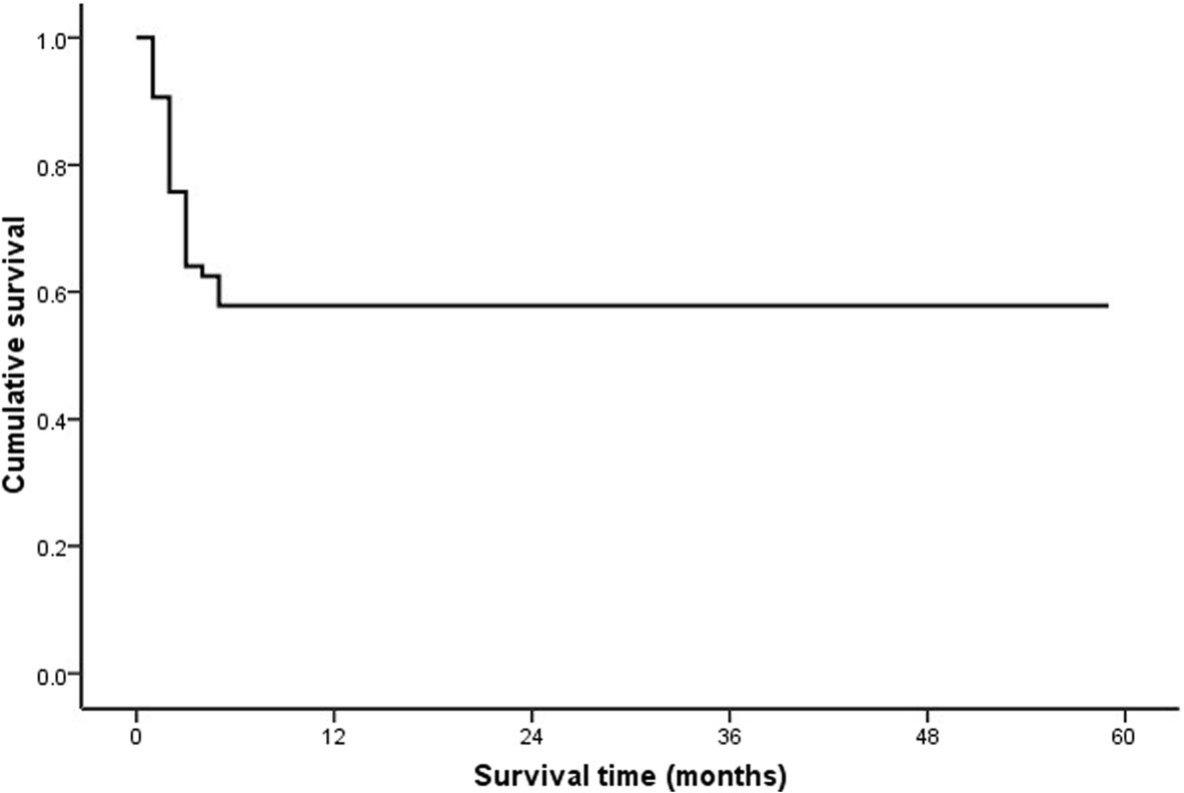
Overall survival in 117 patients with hepatic veno-occlusive disease caused by *Gynura segetum*

In this study, follow-up period ranged from 3 days to 60 months. During the follow-up, 50 patients (43%) died, and causes of death were liver failure (*n* = 19), postoperative multiorgan failure (*n* = 2), cardiovascular disease (*n* = 3), variceal bleeding (*n* = 3), sepsis (*n* = 3), newly developed malignancy (*n* = 5) and combinations of causes (*n* = 9). Information on cause of death could not be retrieved for six patients. Survival rates were 91% (95% confidence interval [CI], 82–95%), 64% (95% CI, 53–69%) and 57% (95% CI, 51–65%) at 1, 3 and 60 months, respectively (Fig. 2).

### Prognostic factors

Univariate analysis revealed that hepatic encephalopathy (*P* = 0.005), total bilirubin (*P* < 0.001), direct bilirubin (*P* = 0.001), albumin (*P* = 0.044), blood urea nitrogen (*P* = 0.022) and treatment (*P* = 0.009) were significantly associated with survival (Table 2).

**Table 2:** Univariate analysis of risk factors associated with survival in 117 patients with hepatic veno-occlusive disease

Variable	Rehabilitation Group (*n* = 67)	Death Group (*n* = 50)	Statistic	*P* value
Age (years)	63.0 (52.3–69.0)	61.5 (48.3–67.8)	*t* = 0.871	0.385
Male/female (%)	67/33	64/36	*χ*^*2*^ = 0.008	0.565
Clinical manifestations, n (%)				
Ascites	67 (100)	49 (98.0)	*χ*^*2*^ = 1.352	0.427
Abdominal distention	66 (98.5)	49 (98.0)	*χ*^*2*^ = 0.044	0.834
Poor appetite	22 (32.8)	26 (52.0)	*χ*^*2*^ = 4.346	0.057
Nausea, vomiting	7 (10.4)	11 (22.0)	*χ*^*2*^ = 2.935	0.120
Malaise	22 (32.8)	17 (34.0)	*χ*^*2*^ = 0.895	0.525
Lower limb oedema	23 (34.3)	23 (46.0)	*χ*^*2*^ = 1.653	0.252
Hepatomegaly	45 (67.2)	37 (74.0)	*χ*^*2*^ = 0.638	0.541
Splenomegaly	22 (32.8)	18 (36.0)	*χ*^*2*^ = 0.127	0.844
Right upper quadrant pain	14 (20.9)	9 (18.0)	*χ*^*2*^ = 0.152	0.815
Weight gain	11 (16.4)	10 (20.0)	*χ*^*2*^ = 0.249	0.634
Encephalopathy	5 (7.5)	14 (28.0)	*χ*^*2*^ = 8.878	0.005
Variceal bleeding	0	3 (6.0)	*χ*^*2*^ = 4.126	0.075
Laboratory tests				
WBC (10^9^/L)	6.1 (5.4–9.3)	6.4 (4.5–8.6)	*t* = 0.171	0.864
RBC (10^12^/L)	4.4 (4.0–4.8)	4.4 (3.3–5.1)	*Z* = 0.933	0.349
PLT (10^9^/L)	115.0 (77.0–148.0)	105 (76–144)	*Z* = 0.676	0.750
ALT (U/L)	69.0 (25.0–201.0)	38.0 (23.0–86.0)	*Z* = 1.040	0.230
AST (U/L)	100.0 (44.0–189.0)	72.0 (38.0–158.0)	*Z* = 0.902	0.389
GGT (U/L)	97.0 (61.5–161.0)	113.1 (49.8–176.6)	*Z* = 0.746	0.635
ALP (U/L)	111.0 (82.5–148.0)	137.0 (84.8–202.0)	*Z* = 1.002	0.267
TB (μmol/L)	26.7 (19.7–36.4)	43.75 (37.0–83.0)	*Z* = 2.163	<0.001
DB (μmol/L)	15.1 (12.8–26.1)	27.9 (21.6–59.4)	*Z* = 1.914	0.001
ALB (g/L)	30.6 (28.9–34.4)	29.8 (27.1–32.5)	*t* = 2.052	0.044
PT (s)	14.8 (12.4–16.5)	15.2 (13.1–18.5)	*t* = −0.774	0.442
BUN (mmol/L)	5.0 (3.8–6.3)	7.0 (3.6–9.51)	*Z* = 1.503	0.022
CR (μmol/L)	71.0 (58.0–84.0)	71.0 (53.5–94.0)	*t* = − 0.088	0.930
CA-125 (u/ml)	224.5 (136.6–430.7)	270.1 (181.9–429.5)	*t* = −0.083	0.935
CA199	11.9 (7.8–25.0)	17.6 (8.3–38.8)	*Z* = 0.672	0.757
Treatment, n (%)				
Non-anticoagulation	9 (13.4)	19 (38.0)	*χ*^*2*^ = 9.498	0.009
Anticoagulation	49 (73.1)	26 (52.0)		
Anticoagulation + TIPS	9 (13.4)	5 (10.0)		

These variables as well as age, sex and platelet count were introduced into a multivariate Cox regression model. Variables were selected using a forward and backward elimination procedure with a significance level of 0.10. The final Cox model showed that total bilirubin (*P* = 0.004), albumin (*P* = 0.024) and hepatic encephalopathy (*P* = 0.018) were independent prognostic markers for survival in this cohort of 117 patients who had complete data for these variables (Table 3).

**Table 3:** Results of the multivariate Cox regression analysis of 117 patients with hepatic veno-occlusive disease

Variable	*P* value	Risk Ratio	95% CI
TB (μmol/L)	0.004	1.006	1.002–1.010
ALB (g/L)	0.024	0.932	0.816–1.063
Encephalopathy	0.018		
Present		3.440	1.240–9.543
Absent			

### Interventions

In this study, 28 of the 117 patients (24%) were managed medically with supportive and diuretic therapeutic approaches such as diuretics, paracentesis, albumin and/or liver function protection only for control of ascites. Conversely, 89 patients of 117 (76%) were treated with anticoagulants in addition to the above mentioned non-anticoagulation therapeutic approaches. None of the patients who were treated with anticoagulant therapy developed complications due to the combination of warfarin and low-molecular-weight heparin, such as severe bleeding. TIPS was performed in 14 patients (12%) during follow-up; five of the patients died because of complications or multiple organ failure. None of the patients received liver transplantation (Table 2).

### Benefit of anticoagulation and TIPS

The use of anticoagulants to prevent thrombosis was found to yield a significant beneficial effect on survival in the current study cohort. Among 28 patients who received non-anticoagulation treatment, 9 (32.1%) were cured, whereas 19 patients (67.9%) died during treatment period. Among a total of 75 patients treated with anticoagulants, 49 patients (65.3%) were cured and 26 patients (34.7%) died (Table 4). The cure rate was higher in the anticoagulation group than in the non-anticoagulation group (χ^2^ = 9.129, *P* = 0.004). The cure rate in the anticoagulation + TIPS treatment group was 64.3%, which was also higher than that in the non-anticoagulation group, albeit without a statistical significance (χ^2^ = 3.938, *P* = 0.096). Furthermore, there was no statistical difference in the cure rates between the anticoagulation group and anticoagulation + TIPS group (χ^2^ = 0.006, *P* = 1.00).

**Table 4:** The effects of different treatment approaches on prognosis of patients with *Gynura segetum*-induced hepatic veno-occlusive disease

Group	n	Clinical Cure		Death	
		n	rate (%)	n	rate (%)
Non-anticoagulation	28	9	32.1	19	67.9
Anticoagulation	75	49	65.3	26	34.7
Anticoagulation + TIPS	14	9	64.3	5	35.7

## Discussion

HVOD, first described by Willmot and Robertson in 1920, is a clinical syndrome characterised by hepatomegaly, weight gain, ascites and jaundice [14, 15]. In Western countries, HVOD is most commonly associated with HSCT and high-dose chemotherapy [3, 16], whereas there is growing concern in developing countries over the use of *Gynura segetum*, which can also cause HVOD. Although hepatic impairment due to conventional pharmaceutical drug use is widely acknowledged, the potential hepatotoxicity of herbal preparations is underestimated because of the public misconception that they are harmless. Importantly, these herbal compounds are commonly used for self-medication without supervision.

Clinically, HVOD diagnosis is usually achieved on the basis of the criteria put forth by the Baltimore and Seattle groups [2, 17], which are based on clinical findings that include painful hepatomegaly, weight gain, hyperbilirubinemia and ascites [14]. The specificity of these two criteria is about 92%; however, their sensitivity is relatively low [10].The current study showed that the most prominent clinical manifestations of *Gynura segetum*-induced HVOD were abdominal distention (98.3%), ascites (99.1%) and hepatomegaly (70%), whereas only about half of the patients exhibited jaundice, and only 19.7% of the patients had right upper quadrant pain. These results are consistent with the previous studies [18]. Thus, the diagnosis in patients of the current study was based on the history of *Gynura segetum* intake, clinical manifestations, imaging results and pathological features. Histological assessment of liver biopsy specimen remains the gold standard for the diagnosis of HVOD; the pathology often includes expansion and congestion of the hepatic sinus, endothelial swelling, wall thickening and incomplete luminal occlusion of the hepatic vein [19]. However, liver biopsy is usually delayed because of extensive ascites, clotting abnormalities and thrombocytopaenia [10]. Recent studies showed that contrast-enhanced computed tomography and magnetic resonance imaging were effective non-invasive methods for the early diagnosis of Tusanqi-induced HVOD and could replace histological examination of the liver in patients with typical clinical data and imaging findings. Patchy enhancement, heterogeneous hypoattenuation in portal phase of the computed tomography or non-homogeneous signal by magnetic resonance imaging are main imaging signs of HVOD [6–8].

In the present study, 5 year survival was 57%, which is higher than those reported from Western countries [20–22]. This difference can be explained by the severe underlying haematological disorders associated with stem cell transplantation in HVOD patients in those studies; development of HVOD following pre-treatment for transplantation is more serious and can easily lead to liver and multiorgan failure and death. In contrast, the patients with *Gynura segetum-*induced HVOD do not necessarily have serious underlying diseases. Moreover, 70% of the current study cohort were treated with anticoagulants, which were reported to contribute to improved HVOD prognosis [23].

The aim of the present study was to assess prognostic determinants of survival in HVOD patients. We identified three important factors that were independently associated with survival: serum bilirubin, serum albumin and encephalopathy. Child–Pugh is a well-known and widely used classification of liver disease. These three prognostic factors identified in the current study are included in the Child–Pugh staging system. Conversely, ascites and prothrombin time did not exhibit a significant impact on survival.

Unlike other common causes of HVOD including high-dose chemotherapy before HSCT, *Gynura segetum*-induced HVOD is unpredictable. It is critical to avoid further contact with the suspicious toxin as soon as possible once symptoms appear or when a definitive diagnosis is reached. Currently, there are no effective therapies for HVOD, and supportive care remains the cornerstone of management, including restriction of water and sodium intake, albumin infusion, abdominal paracentesis and diuretics. Defibrotide is a promising agent with anti-ischaemic, anti-inflammatory and antithrombotic activity [24]; however, the results of defibrotide therapy are conflicting and are not shown to be cost-effective [25]. Our results suggested that anticoagulation therapy significantly increased the cure rate of HVOD, compared with the symptomatic treatment. TIPS is used to relieve portal hypertension and refractory ascites; however, many studies reported that its efficacy in HVOD was poor [26, 27]. Our study also revealed that the cure rate of anticoagulation therapy was comparable with that of anticoagulation therapy in combination with TIPS, with no statistical difference between the two groups, which might be due to the small number of patients receiving the combination treatment. Therefore, future studies with larger cohort sizes are necessary to assess the efficacy and outcomes of combination treatment with anticoagulation therapy and TIPS.

## Conclusions

Major prognostic factors for *Gynura segetum*-induced HVOD were the presence of hepatic encephalopathy and serum bilirubin and albumin levels. Compared with symptomatic treatment, anticoagulation therapy was associated with a significant increased cure rate, but TIPS treatment had no additional effect on improving the cure rate. No uniformly effective treatments are available for HVOD, and the mortality remains high; therefore, prevention of the potent toxicity of *Gynura segetum* is critical. Future studies are necessary to investigate the mechanisms of HVOD to improve the survival rate.

## Abbreviations

CI: Confidence interval
HSCT: Haematopoietic stem cell transplantation
HSOS: Hepatic sinusoidal obstruction syndrome
HVOD: Hepatic veno-occlusive disease
PAs: Pyrrolizidine alkaloids
TIPS: Transjugular intrahepatic portosystemic shunt
